# The IMPROVE-GAP Trial aiming to improve evidence-based management of community-acquired pneumonia: study protocol for a stepped-wedge randomised controlled trial

**DOI:** 10.1186/s13063-017-2407-4

**Published:** 2018-02-05

**Authors:** Elizabeth H. Skinner, Melanie Lloyd, Edward Janus, May Lea Ong, Amalia Karahalios, Terry P. Haines, Anne-Maree Kelly, Melina Shackell, Harin Karunajeewa

**Affiliations:** 1 Department of Physiotherapy, Western Health, 160 Gordon St, Footscray, Victoria 3011 Australia; 2 Department of Physiotherapy, Melbourne School of Health Sciences, University of Melbourne, Parkville, Victoria 3010 Australia; 3 Department of Physiotherapy, School of Primary Care, Faculty of Medicine, Nursing and Health Sciences, Monash University, Frankston, Victoria 3199 Australia; 4 General Internal Medicine Unit, Western Health, Sunshine Hospital, St Albans, Victoria 3021 Australia; 5 Department of Medicine Melbourne Medical School – Western Precinct, University of Melbourne, Sunshine Hospital, St Albans, Victoria 3021 Australia; 6 Centre for Epidemiology and Biostatistics, Melbourne School of Population and Global Health, The University of Melbourne, Parkville, Victoria 3010 Australia; 7 Joseph Epstein Centre for Emergency Medicine Research, Western Health, Sunshine Hospital, St Albans, Victoria 3021 Australia; 8 The Walter and Eliza Hall Institute of Medical Research, G Royal Parade, Parkville, Victoria 3052 Australia

**Keywords:** Community-acquired pneumonia, Randomised controlled trial, Corticosteroids, Antibiotic, Early mobilisation, Malnutrition

## Abstract

**Background:**

Community-acquired pneumonia is a leading worldwide cause of hospital admissions and healthcare resource consumption. The largest proportion of hospitalisations now occurs in older patients, with high rates of multimorbidity and complex care needs. In Australia, this population is usually managed by hospital inpatient general internal medicine units. Adherence to consensus best-practice guidelines is poor. Ensuring evidence-based care and reducing length of stay may improve patient outcomes and reduce organisational costs. This study aims to evaluate an alternative model of care designed to improve adherence to four Level 1 or 2 evidence-supported interventions (routine corticosteroids, early switch to oral antibiotics, early mobilisation and routine malnutrition screening).

**Methods/Design:**

The IMPROVing Evidence-based treatment Gaps and outcomes in community-Acquired Pneumonia (IMPROVE-GAP) trial is a pragmatic, investigator-initiated, stepped-wedge randomised trial. Patients hospitalised under a general internal medicine unit who meet a standard case definition for community-acquired pneumonia will be included. Eight general internal medicine units at two Australian hospitals in a single health service will be randomised using concealed allocation to: (i) usual medical, nursing and allied health care delivered according to existing organisational practice or (ii) care supported by a dedicated “community-acquired pneumonia service”: a multidisciplinary team deploying algorithm-based implementation of a bundle of the four evidence-based interventions. The primary outcome measure will be length of hospital stay. Secondary outcome measures include inpatient mortality, 30 and 90 day readmission rates and mortality and health-service utilisation costs. Protocol adherence will be measured and reported, and serious adverse events (rates of hyperglycaemia requiring new insulin; falls during mobilisation) will be collected and reported.

**Discussion:**

IMPROVE-GAP represents an important and unique precedent for testing a new service-delivery model for improving compliance with a number of evidence-based interventions. Its stepped-wedge randomised controlled trial design provides a means to address some significant ethical, organisational and other methodological challenges to evaluating the effectiveness of health-service interventions in complex hospital populations. The new service-delivery model will effectively be fully implemented by trial completion, facilitating rapid, seamless translation into practice should care outcomes be superior. This trial is currently recruiting.

**Trial registration:**

ClinicalTrials.gov, NCT02835040. Prospectively registered on 22 May 2016.

**Electronic supplementary material:**

The online version of this article (doi:10.1186/s13063-017-2407-4) contains supplementary material, which is available to authorized users.

## Background

Community-acquired pneumonia is the second leading cause of mortality worldwide [1] and mortality in hospitalised patients is as high as 13% [2]. In Australia, community-acquired pneumonia is responsible for more hospital admissions than any other single illness (61,000 hospital admissions per year) [3] and incurs direct healthcare costs of more than AU $300 million annually [4]. Prolonged length of stay can increase organisational costs and is strongly associated with adverse patient outcomes, including loss of function due to de-conditioning [5] and a higher incidence of hospital-acquired adverse events, such as hospital-acquired infections, intravascular-device associated complications and antibiotic-related side effects [6–8]. In the modern era, in developed countries, a majority of the population hospitalised for community-acquired pneumonia are elderly and have a high prevalence of multimorbidity, which is independently associated with mortality [9]. This group also incurs the highest costs, owing to longer hospitalisations, higher readmission risks and poor functional outcomes. Multimorbidity also increases complexity of care, which makes it more difficult to maintain compliance with evidence-based guidelines. In Australia, acute unplanned non-surgical hospital admissions of multimorbid patients are largely managed by general internal medicine units, who therefore now manage the largest proportion of patients hospitalised with community-acquired pneumonia. With population ageing, the elderly and highly multimorbid population treated by general internal medicine units is likely to constitute the bulk of Australia’s future health-service burden for community-acquired pneumonia [10].

We have identified four key interventions (adjunctive corticosteroids, oral antibiotics, early mobilisation and routine malnutrition screening) that are now supported by Level 1 or 2 evidence demonstrating improvement in clinical outcomes in patients with community-acquired pneumonia.

At the time this trial was designed, a body of evidence had accrued to support the efficacy of adjunct corticosteroids, including results from two large randomised controlled trials (RCTs) showing that they (1) reduced treatment failure in severe community-acquired pneumonia [11] and (2) shortened time to clinical stability and time to effective hospital discharge without an increase in adverse complications [12]. Although there is a slightly higher risk of hyperglycaemia, this can be effectively treated with insulin with no long-term effects [12, 13]. Meta-analyses had also confirmed these findings and demonstrated an overall lower rate of complications in corticosteroid-treated patients, including a reduction in the need for vasopressors or mechanical ventilation in patients with community-acquired pneumonia routinely prescribed corticosteroids [14, 15]. Early mobilisation safely and effectively reduces length of stay [16], as does an early switch from intravenous to oral antibiotics [17]. A recent randomised trial of both these interventions found that length of stay was shortened by two days compared with standard care [18]. In a recent meta-analysis of malnourished medical inpatients (including those admitted with community-acquired pneumonia), systematic screening for malnutrition risk and targeted nutritional therapy reduced non-elective readmission rates [19].

**Fig. 1:**
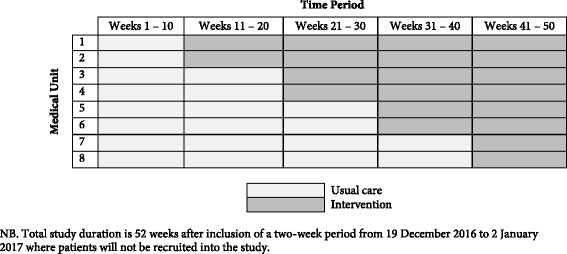
Stepped-wedge rollout of community-acquired pneumonia service by general internal medicine unit

Despite this high-level evidence, these interventions are poorly or not routinely deployed in routine clinical practice [20]. Therefore, they represent areas where there is significant scope to improve the translation of evidence into clinical practice, demonstrating a clear “evidence-practice gap”. The notoriously poor adherence to consensus guidelines for community-acquired pneumonia is consistent with a broader general problem of widespread delays and inconsistency in translation of evidence into healthcare practice in a variety of fields [21]. This results in poorer patient outcomes and a greater healthcare and societal burden. Therefore, improving this “evidence-practice gap” has been recognised as a leading priority for the medical research establishment in Australia and elsewhere [22]. Innovative health-service approaches, including alternative models of care, will be required to bridge this gap and it will be important that their effectiveness is measured in a suitably robust fashion. Evaluations should therefore: (1) be conducted in appropriately “real-world” settings in representative populations that enable generalisability throughout the health system and (2) be appropriately statistically powered and designed in a way that minimises potential for confounding and bias that could lead to misleading conclusions regarding the presence, or lack thereof, of a real impact of the intervention. However, in practice there are very important and difficult methodological challenges to designing evaluations of this type, especially for conditions like community-acquired pneumonia, which now largely manifests in patients who may be difficult to enrol into studies utilising conventional research designs. In particular, high rates of cognitive impairment, confusion or drowsiness, general frailty, severe or life-threatening illness and (in populations such as ours) high proportions from culturally and linguistically diverse backgrounds are extremely challenging to enrol into interventional studies based on individual randomisation and consent [23, 24]. This has resulted in a very well-documented phenomenon, where the usefulness of clinical trial data comes into question, owing to its poor generalisability [25, 26]. Moreover, health-service interventions are fundamentally designed to be deployed on a large scale (e.g. organisation-wide), meaning that deployment on a small scale (e.g. based on individual randomisation) is impractical. Therefore, it is more appropriate to utilise designs where the unit of randomisation is larger – ideally based on a practical sub-division of the existing health system that can then be used as the unit for randomisation to determine which cluster does or does not receive the intervention. The relatively recent development of stepped-wedge study designs may provide a very effective tool in this context. Stepped-wedge studies are effectively a type of cluster RCT. However, unlike conventional cluster RCTs, in stepped-wedge approaches, after a baseline period during which none of the clusters receives the intervention, the intervention is then progressively “rolled out” in constant increments in several clusters over time so that, by study conclusion, all clusters are receiving the intervention (see Fig. 1). This design is therefore analogous to an “upscaling” that effectively mimics the way an intervention might be deployed in practice and is therefore particularly well-suited to implementation and health-service research [27, 28]. Statistical analysis principles have now been established to ensure that the effects of cluster and variation in outcomes across time periods are appropriately modelled and accounted for when developing estimates of treatment effect from these designs [29]. This approach has significant logistic, financial and ethical advantages over conventional cluster and individual RCT approaches, particularly where collected outcomes are part of usual care, minimising additional burden to researchers and participants [27, 28, 30, 31]. On conclusion of the study, all clusters will be receiving the intervention (implemented across the whole service), which could then be continued indefinitely if it proves effective. This approach allows a seamless transition to local implementation and can reduce the time to clinical translation significantly when compared with conventional approaches [21].

A key barrier to translation involves changing clinician behaviour; we therefore hypothesise that an effective way to meet the challenge of improving compliance with a number of evidence-based interventions simultaneously in a complex patient group, is to utilise an independent syndrome-based clinical service for community-acquired pneumonia, analogous to those applied in other areas (e.g. stroke services) [32]. Our proposed community-acquired pneumonia service would have core responsibility for ensuring comprehensive and rigorous current evidence-based best practice by recommending that clinical teams align treatment with a standardised set of management algorithms incorporating interventions supported by at least Level 2 evidence. This novel service-delivery approach therefore represents the intervention being tested in our stepped-wedge study.

### Trial objectives

The primary objective of the IMPROVing Evidence-based treatment Gaps and outcomes in community-Acquired Pneumonia (IMPROVE-GAP) trial is to estimate the effect that a translation community-acquired pneumonia service delivering four evidence-based interventions has on length of hospital stay, when compared with usual hospital care. Secondary objectives are to evaluate the effect of the community-acquired pneumonia service on inpatient mortality, 30 and 90 day readmission rates and mortality, and health-service costs.

## Methods/Design

### Trial design

The IMPROVE-GAP trial is a pragmatic, investigator-initiated, stepped-wedge randomised controlled clinical effectiveness study. This protocol is reported according to the SPIRIT checklist (Additional file 1) [33].

### Trial setting

The two participating hospitals (Footscray Hospital and Sunshine Hospital) are both part of Western Health, a health service that services a population of approximately 700,000 in inner metropolitan Melbourne, Victoria, Australia. Footscray and Sunshine Hospitals are tertiary metropolitan primary referral hospitals with 260 and 510 beds, respectively. Both are publicly funded, university-affiliated teaching hospitals. General internal medicine services manage the largest proportion of patients with community-acquired pneumonia in our health service, with 47% of admissions for community-acquired pneumonia managed by general internal medicine from 2012 to 2013 (average age, 75 years, with proportions with at least 1, 2 or 3 active comorbidities of 70%, 43% and 27% respectively). Western Health has a total of eight general internal medicine units split evenly across the two participating hospitals, which will constitute the eight separate clusters used in the stepped-wedge design.

### Eligibility and exclusion criteria

All adult patients (aged ≥ 18) admitted to Western Health general internal medicine units will be eligible to participate. Only participants who meet a standardised community-acquired pneumonia case definition [34] will be included. The conventional case definition, as used in recent studies of community-acquired pneumonia [12, 35], is as follows:

1. New infiltrate on chest X-ray
2. The presence of at least one of the following acute respiratory symptoms, signs or laboratory test results: cough, sputum production, dyspnoea, core body temperature ≥ 38.0 °C, auscultatory findings of abnormal breathing sounds or rales, leucocyte count > 10,000 /μl or < 4000 /μl

Patients with the following criteria will be excluded: (1) decision to implement palliative care on admission; or (2) enrolment in another clinical trial.

### Ascertainment, enrolment and waiver of consent

Ascertainment will be conducted prospectively to identify eligible participants in real time ensuring employment of identical methods of ascertainment for both control and intervention groups. A community-acquired pneumonia service team member will attend each general internal medicine morning clinical handover meeting to identify new admissions with possible respiratory infection from the previous 24 hours. Either the chief medical registrar or treating medical registrar will subsequently review the medical record and chest X-ray (for evidence of a new infiltrate, as per requirements of the standardised case definition) of potential participants to assess eligibility. Those meeting this case definition [34] and eligibility criteria will be enrolled. In situations where the chest radiograph or community-acquired pneumonia diagnosis are not definitive, the treating medical consultant will determine eligibility.

The study is conducted in accordance with the Declaration of Helsinki and was approved by the hospital’s institutional review board (Melbourne Health Human Research Ethics Committee [protocol reference, MH2016.014]). The trial was prospectively registered on 22 May 2016, at www.ClinicalTrials.gov (NCT02835040). Importantly, our supervising human research ethics committee granted approval for waiver of consent for enrolment on the basis that (1) the intervention represented a systematic implementation of standardised evidence-based best practice, rather than the testing of an unproven novel treatment, (2) study outcomes are already routinely collected by the health service and (3) the study’s rationale and proposed methods met each of the Australian National Health and Medical Research Council’s criteria for appropriate use of a waiver of consent [36]. This means that individual consent is not required and that all admitted patients who satisfy the inclusion and exclusion criteria will be enrolled during the study period. This will ensure that a representative and generalisable study population is enrolled. *A priori,* plans for communicating protocol modifications were deemed unnecessary, as alterations to the study design are not feasible in a stepped-wedge trial and we did not foresee a need for amendments. Any major amendments will be documented on trial registries and reported in the final manuscript. Ethical approval was also sought and gained for storage of deidentified participant data in a databank, which may be accessed for future studies, pending future additional and separate human research ethics committee submissions. All investigators will have access to the final trial dataset, and no contractual agreements limit such access.

A nested sub-study, to be described separately, is planned by co-enrolling participants in the intervention arm (anticipated *n* = 40). This study will require separate ethical submission and approval with individual written informed consent sought from these participants for collection of ancillary data (relating to patient-reported outcomes) and biological specimens (nose, throat swabs and sputum samples) for reporting separately from the main study. This separately reported sub-study will also utilise data collected through IMPROVE-GAP’s trial procedures and routine data collection in this sub-group.

### Randomisation and allocation

As previously described (Fig. 1), the institution has four general internal medicine units at each of two sites (Footscray and Sunshine); therefore, a total of eight units constitute the eight separate clusters used in the stepped wedge. Units will be assigned to usual care or intervention (community-acquired pneumonia service). The order of community-acquired pneumonia service intervention rollout will be randomised (two general internal medicine units at a time across eight general internal medicine units). The random allocation sequence will be generated by a statistician not involved with the study, using Stata version 13.1 (StataCorp. 2013. Stata Statistical Software: Release 13. College Station, TX: StataCorp LP).

### Stepped-wedge rollout and timeline

After a 10 week period of baseline data collection (during which all eight units receive conventional care), the community-acquired pneumonia service intervention will be rolled out, with two general internal medicine units each assigned to commence the intervention at either 11, 21, 31 or 41 weeks (assignment by pre-determined randomisation schedule). All eight general internal medicine units will receive the intervention for the final 10 weeks of the study; and patients will not be recruited into the study for two weeks (19 December 2016 to 2 January 2017), to allow for bed closures and staff absence over the Christmas holiday period. During this time, interventions for those already enrolled will continue as per the randomisation schedule. Therefore, the total recruitment period of the study will be 50 weeks (Fig. 1).

**Fig. 2:**
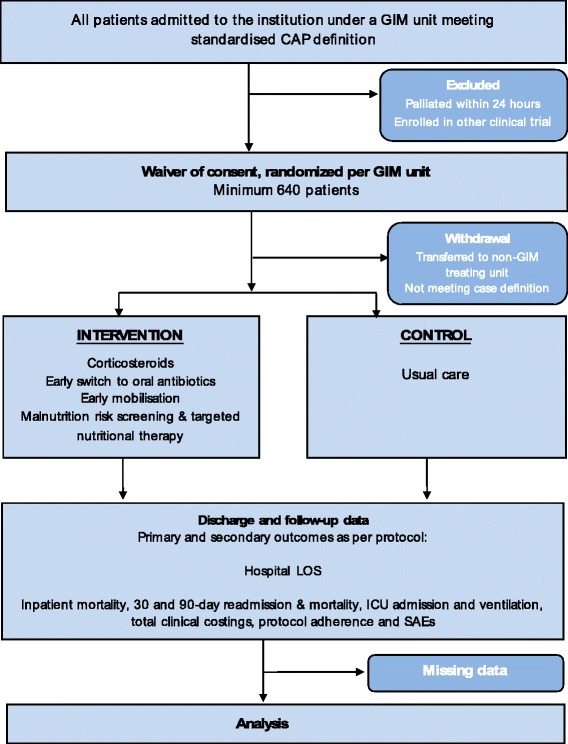
CONSORT flow diagram of IMPROVE-GAP. CAP, community-acquired pneumonia; GIM, general internal medicine; ICU, intensive care unit; LOS, length of stay; SAE, serious adverse event

**Fig. 3:**
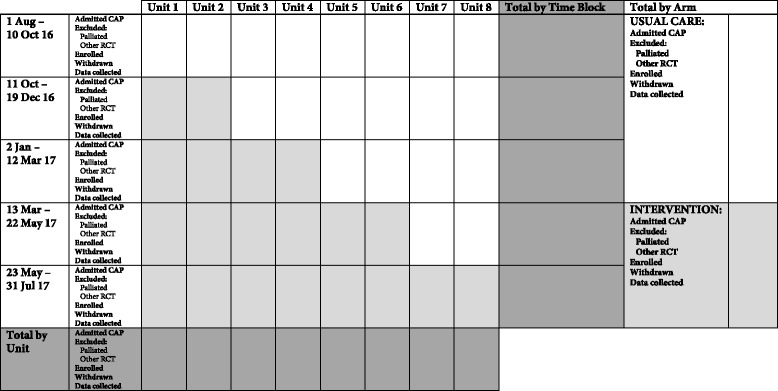
Summary diagram of stepped-wedge design. CAP, community-acquired pneumonia; RCT, randomised controlled trial

**Fig. 4:**
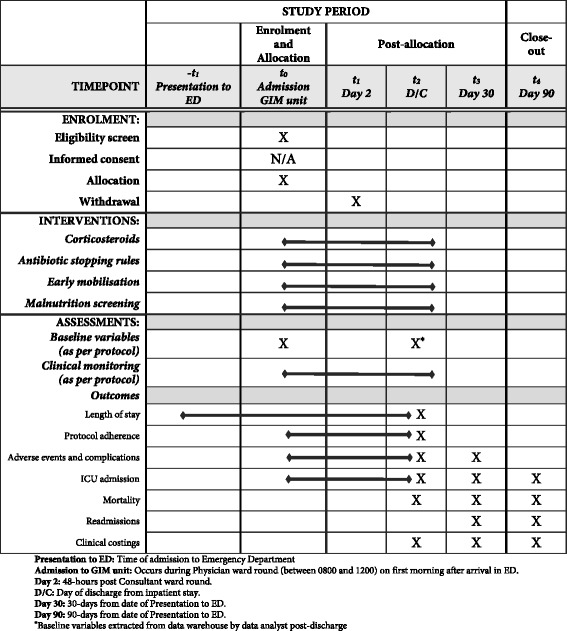
Schedule of enrolment, interventions and assessments (as per SPIRIT [33]). D/C, discharge; ED, emergency department; GIM, general internal medicine; ICU, intensive care unit

The CONSORT diagram of the IMPROVE-GAP trial is presented in Fig. 2, along with an additional diagram that more clearly presents the recruitment phase of the study (Fig. 3). We also present a detailed schedule of enrolment, intervention and assessments as per SPIRIT (Fig. 4) [33].

### Trial intervention

The detailed outline and description of the two study groups (intervention and control) are provided according to the Template for Intervention Description and Replication (TIDieR) criteria in Table 1 [37].

**Table 1:** Description of intervention and usual care groups according to the Template for Intervention Description and Replication (TIDieR) [37]

TIDieR criterion	Intervention	Usual care
Item 1. Brief name: Provide the name or a phrase that describes the intervention	Community-acquired pneumonia service	Usual inpatient hospital care
Item 2. Why: Describe any rationale, theory or goal of the elements essential to the intervention	A large RCT and meta-analysis [12, 14] demonstrated faster clinical recovery and shorter length of stay with adjunct corticosteroids without significant adverse events. Routine adjunctive corticosteroid is now widely supported though as yet not consistently deployed. Early switch to oral antibiotics guided by a set of well-defined basic clinical and laboratory criteria [17] also reduces length of stay. Recently, a RCT incorporating both measures demonstrated a length of stay reduction of 2 days compared with standard care [18]. Early mobilisation safely and effectively reduces length of stay when applied appropriately [16]. Routine screening of medical inpatients for malnutrition and appropriate targeted nutrition therapy can reduce unplanned readmissions [19]	Usual inpatient hospital care will be delivered as per underlying usual care rationale, theories and goals of community-acquired pneumonia management
Item 3. What (materials): Describe any physical or informational materials used in the intervention, including those provided to participants or used in intervention delivery or in training of intervention providers	Patient information materials will not apply as a waiver of consent is sought. Intervention providers will be given an intervention algorithm tool to prompt safe, systematic and appropriate initiation of the four evidence-based interventions	Nil additional to usual care
Item 4. What (procedures): Describe each of the procedures, activities or processes used in the intervention, including any enabling or support activities	The community-acquired pneumonia service will apply a set of protocols to ensure rigorous application of interventions each with proven efficacy including: 1.Routine prescription of 50 mg prednisolone for 7 days (following checklist exclusion of those with contraindications) [12, 14] 2.Constrained parenteral antibiotic duration (using pre-defined ‘stopping rules’) [17, 18] 3.Physiotherapy-led early (< 24 h) mobilisation [16] 4.Routine malnutrition screening and implementation of an appropriate nutrition therapy intervention as indicated [19, 41]	During the non-interventional control periods (as determined by the stepped-wedge rollout schedule) patients with community-acquired pneumonia will receive conventional care by the usual treating general internal medicine team: currently, 43% receive corticosteroids, 63% physiotherapy (median time to initiation 2 days), 65% guideline-compliant antibiotics [4]. No parenteral antibiotic stopping rules are in place (median 3 days). 72% of inpatients at Western Health currently receive routine malnutrition screening
Item 5. Who provided: For each category of intervention provider (for example, psychologist, nursing assistant), describe their expertise, background and any specific training given	Relevant members of the general internal medicine multidisciplinary team (doctors, nurses, physiotherapists and dietitians) will deliver the community-acquired pneumonia service intervention. Clinicians treating patients in the intervention arm of the study will be given an education package outlining current evidence-based practice guidelines and the treatment protocols to be applied. Intervention arm clinicians will also have access to decision-support algorithms to promote consistent application of the protocols	The general internal medicine multidisciplinary team will deliver usual care
Item 6. How: Describe the modes of delivery (such as face to face or by some other mechanism, such as internet or telephone) of the intervention and whether it was provided individually or in a group	Face-to-face individual intervention	Face-to-face individual intervention
Item 7. Where: Describe the types of location where the intervention occurred, including any necessary infrastructure or relevant features	Acute hospital wards; patients under the care of the general internal medicine unit	Acute hospital wards; patients under the care of the general internal medicine unit
Item 8. When and how much: Describe the number of times the intervention was delivered and over what period of time, including the number of sessions, their schedule, and their duration, intensity or dose	Daily during acute hospital admission	During acute hospital admission at the discretion of treating medical, allied health and nursing clinicians
Item 9. Tailoring: If the intervention was planned to be personalised, titrated or adapted, then describe what, why, when and how	The four best-practice interventions will aim to be delivered to all patients meeting the inclusion criteria, except in the case of specific contraindication to an intervention as outlined in this protocol. The protocol for each of the interventions also outlines circumstances where treatment can be individualised. General internal medicine consultants may vary their provision of care given difficulties in delivering rigid protocols in a healthcare setting and their perspective of the importance of delivering individualised medicine; however, non-adherence and the reasons for it will be measured and reported for any deviations in the protocols	At the discretion of the treating medical team and allied health clinicians
Item 10. Modifications: If the intervention was modified during the course of the study, describe the changes (what, why, when, how)	Not applicable in protocol	Not applicable in protocol
Item 11. How well (planned): If intervention adherence or fidelity was assessed, describe how and by whom; if any strategies were used to maintain or improve fidelity, describe them	Patient proportions receiving: i) Corticosteroids ii) Pre-defined intravenous antibiotic stopping rules iii) Early mobilisation iv) Malnutrition Screening Tool application and appropriate nutrition therapy Aim for relevant proportions to exceed 70% for interventions (i) and (ii), and 85% for (iii) and (iv). Protocol adherence rates will be available to the project coordinator throughout the study, allowing high protocol non-compliance rates to be addressed in a timely fashion. Fidelity will be maximised by the daily interaction with community-acquired pneumonia team which promotes and monitors protocol adherence	Patient proportions receiving: i) Corticosteroids ii) Pre-defined intravenous antibiotic stopping rules iii) Early mobilisation iv) Malnutrition Screening Tool application and appropriate nutrition therapy. Anticipate existing data on proportions to be maintained
Item 12: How well (actual): If intervention adherence or fidelity was assessed, describe the extent to which the intervention was delivered as planned	Not applicable in protocol	Not applicable in protocol

The trial intervention is a 7 days/week evidence-based model of service (community-acquired pneumonia service) that comprises a multidisciplinary physiotherapist-led team (including medical, dietetics collaboration) that will conduct daily rounds of all patients managed by general internal medicine units assigned to the intervention.

The community-acquired pneumonia service will provide four protocolised evidence-based interventions using custom-designed decision-support algorithms. Patients with specific contraindications to corticosteroid therapy or early mobilisation will not receive these interventions. The four interventions are as follows.

#### Intervention 1 – Corticosteroids

Treating clinicians will be advised to prescribe 50 mg prednisolone daily for 7 days. See Additional file 2 for details of specific contraindications for corticosteroid therapy.

Patients with diagnosed diabetes will be routinely monitored for hyperglycaemia (see Data monitoring section) and managed at the discretion of the treating team in accordance with existing institutional procedures and practices. All admitted patients have blood glucose levels measured in the emergency department, and ongoing random blood glucose monitoring may also be implemented for other patients thought to be at risk of hyperglycaemia at the discretion of the treating team. Any variation to the dose and duration, such as use of tapered dosage, and time from admission until first dose will also be recorded. Alternative medical reasons given for non-prescription of corticosteroids, or reduced duration of administration, will be noted.

#### Intervention 2 – Early switch to oral antibiotics

Guideline-consistent prescription of initial antibiotic therapy and constrained parenteral antibiotic duration (using pre-defined stopping rules) will be provided in the best-practice group [17, 18].

Current institutional general internal medicine antibiotic prescribing practice aims to follow an institutional guideline based on the Australian Antibiotic Guidelines [38]. Patients are stratified as having mild, moderate or severe disease using standardised pneumonia severity criteria. Our institution uses the CORB score, based on its ease and feasibility of use, and it having the highest predictive value for poor outcomes [10]. Based on CORB, most inpatients receive either intravenous penicillin and doxycycline, or roxithromycin (mild or moderate disease), or ceftriaxone and azithromycin (severe disease).

Patients will be switched from intravenous to oral therapy according to the specific criteria for clinical improvement, which will be assessed on a daily basis by the community-acquired pneumonia service using a standardised checklist (also commonly referred to as ‘stopping rules’, see Additional file 2). Essentially, for switching to oral antibiotics, patients must be able to maintain oral intake, have stable vital signs, stable SpO_2_ and no evidence of septic metastases or major exacerbated comorbidities [18].

Any reason for alternative antibiotic selection (such as allergy or advice from an infectious diseases consultant) will be noted.

#### Intervention 3 – Early mobilisation

Physiotherapist-led early (<24 h) and progressive mobilisation will be provided daily, and is defined as movement out of bed with change from horizontal to upright position for at least 20 min during the first 24 hours of admission to a general internal medicine unit (a minimum score of 2 on the Intensive Care Unit Mobility Scale [39]), and progressive movement each subsequent day [16]. See Additional file 2 for intervention details, including specific contraindications and stopping criteria for daily mobilisation.

Exercise tolerance (total distance mobilised before requiring a rest, and total distance mobilised during the session) will be recorded for each session, as will any reason why early or progressive movement is not achieved and any adverse events during physiotherapy sessions.

#### Intervention 4 – Malnutrition risk screening with targeted nutritional therapy

Standardised malnutrition screening, using the Malnutrition Screening Tool [40] and measurement of patient body weight (in kilograms) will be completed within 24 hours of admission to a general internal medicine unit. The Malnutrition Screening Tool score will guide implementation of nutritional therapy as follows [41]:

- 0–1: No nutrition therapy intervention indicated
- 2–3: Initiation of a high-energy high-protein diet by nursing staff
- 4–5: Referral to the institutional Dietetics Service for urgent review and implementation of an individually tailored malnutrition intervention

Reasons for non-compliance with the malnutrition screening protocol will be noted.

#### Control group

During the non-interventional control periods (as determined by the stepped-wedge rollout schedule), patients admitted with community-acquired pneumonia under eligible general internal medicine units will receive usual care at the discretion of the treating general internal medicine multidisciplinary team. Ultimate responsibility for treatment decisions is generally made by a supervising general internal medicine consultant physician, who reviews patients on the morning after admission and on supervisory ward rounds conducted three times per week. Junior medical staff (registrar level) are also involved in day-to-day medical decision-making but allied health practitioners (physiotherapists and dieticians) are generally only involved if there has been a formal referral process initiated by a member of the treating medical or nursing team.

Pre-trial standard care at the study institution (auditing conducted in 2015) consisted of the following proportions of patients with community-acquired pneumonia receiving: corticosteroids (43%), a guideline-compliant antibiotic (65%) and physiotherapy (including early mobilisation) (63%; median time to initiation, two days). No parenteral antibiotic stopping rules are in place (median duration 3 days), while malnutrition risk screening in inpatients across all health-service patients is 72%.

All other aspects of patient care, including fluid delivery, use of lines and drains, general nursing care and discharge planning, will be provided at the discretion of the general internal medicine teams, as per routine institutional practice or protocols.

### Blinding

Clinicians providing patient interventions cannot be blinded, although participants are effectively blinded, owing to the waiver of consent. All primary and secondary outcome measures are routinely collected within the organisation by staff not involved in delivery of the intervention, who will be unaware of participant group allocation. There is therefore little or no potential risk of bias in outcome assessments. A statistician blinded to the allocation sequence will perform data analysis.

### Withdrawal from trial

Participants will be withdrawn if they are transferred to another unit (e.g. specialist respiratory, cardiology or surgical) or another health service within 48 hours of admission, or are no longer considered to meet the community-acquired pneumonia case definition on consultant review. All withdrawals and reasons will be reported.

### Primary outcome measure

The primary outcome measure will be hospital length of stay, calculated from arrival in the emergency department until discharge from the health service, based on date and time (note that data will be collected in minutes but converted to days). This information is routinely collected at the institution for all inpatients.

### Secondary trial outcome measures

Secondary outcome measures comprise routinely collected institutional data and include:

1. Inpatient mortality
2. 30 day and 90 day readmission rates and mortality
3. Admission to the intensive care unit from inpatient wards and requirement for and duration of mechanical ventilation
4. Total individual per-separation hospital clinical costing, as determined by the institutional dedicated clinical costing unit using the Power Performance Manager™ software platform (Power Health Solutions, Adelaide, Australia)
5. Protocol adherence (proportion of evidence-based treatments delivered in each group; proportion of patients receiving the whole bundle; reasons for protocol adherence failure)
6. Serious adverse events (including the incidence of in-hospital hyperglycaemia requiring new insulin prescription, adverse drug reactions where it is required that the drug use is stopped, falls or clinical deterioration requiring urgent medical review during early mobilisation), other adverse events and clinical complications (Table 2) occurring prior to admission, during inpatient stay or requiring re-presentation to hospital within 30 days.

**Table 2:** Reporting of serious adverse events, adverse events and complications

Complication group	Complication
Pneumonia-associated complications	Respiratory failure requiring intubation
	Hypotension requiring vasopressors
	Empyema *Defined as: documented in registrar admission assessment, radiologist chest X-ray report or medical discharge summary*
	Acute respiratory distress syndrome *Defined as: documented in registrar admission assessment, radiologist chest X-ray report or medical discharge summary*
	Pleural effusion *Defined as: diagnosed on radiologist chest X-ray report*
	Increased confusion from baseline
Other adverse events	Death from any cause
	Fall with fracture
	Cardiac decompensation *Defined as: documented in registrar admission assessment, radiologist chest X-ray report or medical discharge summary*
	Cardiac event *Defined as: diagnosed episode of cardiac ischaemia or new cardiac arrhythmia*
	Acute stroke *Defined as: documented in registrar admission assessment, radiologist report or medical discharge summary*
	Thromboembolic event *Defined as: pulmonary embolus or deep vein thrombosis documented in registrar admission assessment, radiologist report or medical discharge summary*
	Confirmed or suspected gastrointestinal bleeding
Adverse events in patients receiving corticosteroids	Adverse drug reaction where it is required that the drug is stopped (percentage of total number receiving burst corticosteroid dose)
	In-hospital hyperglycaemia requiring new insulin prescription (percentage of known diabetics)
Adverse events compatible with antibiotic use	Adverse drug reaction, where it is required that the drug use is stopped
Adverse events in patients receiving early mobilisation	Falls during physiotherapy (percentage of total physiotherapy sessions delivered)
	Clinical deterioration during physiotherapy requiring urgent medical review (percentage of total physiotherapy sessions delivered) (see Additional file 2)
	Mobilisation ceased owing to sustained observations outside target range (percentage of total physiotherapy sessions delivered) (see Additional file 2)

### Protocol adherence

Protocol adherence to each component of the intervention will be measured and reported as follows.

### Intervention 1 – Corticosteroids

1. Prescription of 50 mg prednisolone daily (or equivalent dose of hydrocortisone or other corticosteroid) within 36 hours of arrival at the emergency department
2. Minimum 7 day duration of corticosteroid prescription

### Intervention 2 – Early switch to oral antibiotics

1. Adherence to intravenous antibiotic stopping rules, where the switch to oral therapy is made within 24 hours of the patient meeting the criteria for clinical improvement

### Intervention 3: Early mobilisation

1. Completion of first session of early mobilisation with a physiotherapist within first 24 hours of admission to a general internal medicine unit, and achieved minimum physical activity of sitting out of bed > 20 minutes
2. On > 70% of eligible admission days (early mobilisation not contraindicated) progressive movement is achieved in a physiotherapy session
3. Patient movement either upright in bed or to the commode for toileting alone will be considered insufficient [16]

### Intervention 4: Malnutrition risk screening and targeted nutrition therapy

Adherence will be measured by:

1. Calculation of Malnutrition Screening Tool score within 24 hours of admission to a general internal medicine unit
2. Implementation of appropriate nutrition therapy in response to Malnutrition Screening Tool score (none, high-energy high-protein diet or dietician referral and review within 24 hours)

### Data collection (see Fig. 4)

#### At enrolment (baseline measures)

Demographic and clinical characteristics will be recorded by collection of the following variables (directly from the patient or the medical record): site, patient age and sex, admitting medical unit, eligibility criteria (age, chest X-ray findings, cough, dyspnoea, temperature, chest auscultation findings, peripheral blood leucocyte count), vital signs on admission (temperature, heart rate, blood pressure, respiratory rate, pulse rate, confusion), estimated glomerular filtration rate, residential care status (independent living, supported accommodation, aged care facility), pre-morbid mobility, function and exercise tolerance, CORB score (derived from confusion, oxygenation, respiratory rate and blood pressure), any relevant comorbidities (e.g. diabetes, immunosuppression, adrenal insufficiency, chronic obstructive pulmonary disease, chronic cardiac failure, malignant process), number of routine medications used by the patient, baseline corticosteroid dose (if any), baseline insulin usage, relevant drug allergies, enrolment in a concurrent inpatient research trial, any decision to initiate palliative care on admission, any adverse events or complications that occur prior to enrolment.

#### Daily during admission (clinical monitoring)

Circumstances necessitating withdrawal of a participant from the study after enrolment but within 48 hours of admission to a general internal medicine unit will be recorded.

The following variables will be collected from the medical record and reported for all enrolled patients: any serious adverse events, adverse events or clinical complications (see Table 2), any decision to palliate, adherence to protocol for Interventions 1 to 4 (as listed previously).

Daily vital signs (SpO_2_, supplementary oxygen requirements, heart rate, respiratory rate, blood pressure, temperature) will be measured every 4 hours as per routine clinical care and used to ascertain the switch to oral from intravenous antibiotics.

Blood glucose readings as clinically indicated and requested by treating team, and oral intake status (ability to tolerate oral medications and absence of gastrointestinal problems that may affect drug absorption) will also be reviewed daily.

#### At discharge (clinical and outcome measures)

On acute hospital discharge via review of the medical record, the admission chest X-ray report (as reported by the radiologist), along with other diagnostic test results (respiratory PCR, legionella urinary antigen, sputum and blood cultures, serology) will be recorded.

The following variables will be extracted directly from the institutional data warehouse and linked to the study dataset by unique identifier (admission episode number): age, sex, marital status, language status, primary ICD-10 discharge code and allocated diagnosis-related group, ICD-10 co-morbidity groupings used in the Charlson’s Co-morbidity Index (derived using an existing algorithm that interrogates ICD-10 coding data), intensive care unit length of stay and duration of mechanical ventilation, total length of stay measured in days (to three decimal places) and broken down to emergency department, inpatient ward, rehabilitation or sub-acute care and hospital in the home), inpatient mortality and total individual clinical costings.

#### At 30 and 90 days following presentation to the emergency department (outcome measures)

Mortality, institutional readmission within 30 and 90 days and total institutional costs incurred at 30 and 90 days will also be extracted from the institutional data warehouse and linked to the study dataset as described previously.

Any adverse event or complication (as defined in Table 2) that requires re-presentation to hospital noted in the electronic medical record within 30 days of initial arrival in the emergency department will be recorded.

### Sample size

Using a stepped-wedge cluster-randomised trial with eight general internal medicine units, over five time periods or steps (i.e. baseline plus four intervention steps), a sample size of 80 patients per general internal medicine unit (640 patients total) will be sufficient to detect a clinically important decrease in the proportion of patients with a length of stay greater than the median length of stay from 36% to 20%, assuming an intra-cluster correlation of 0.01, with 75% power and a 5% significance level [42]. This will also be sufficient to estimate absolute and relative reductions in mean length of stay between the intervention and control periods.

Western Health’s 2014–2015 admission data showed that the total number of annual separations with an ICD-10 diagnosis code of pneumonia (J12–J18, excluding J35) admitted to a general internal medicine unit at either Footscray or Sunshine Hospital was 1008. It is therefore expected that there will be an adequate number of admissions during the 50-week study period to satisfy the sample size requirements, allowing for an exclusion rate of 20%.

### Data management

All data will be collected through existing routine processes and captured in the patient electronic medical record stored on the password-protected hospital admission information systems (iPM / EDIS v.EDISAPAC 15.1.0 [CSC, Tysons, Virginia USA] and Bossnet [Core Medical Solutions, Rose Park, South Australia]). Data from the electronic medical record will be extracted by study investigators where required throughout admission and after discharge from hospital.

Data will be extracted from iPM and EDIS systems after the patient has been discharged and then separately entered into a dedicated REDCap (research electronic data capture) database [43] hosted at the University of Melbourne, Melbourne, Australia. An investigator will randomly audit data collection of one patient per each ten recruited to ensure data quality and data entry will be randomly checked and cleaned by an investigator, either a chief investigator or an investigator supervised by a chief investigator. On publication of the trial, the database will be made available for independent analysis pending the necessary ethical approvals if requested.

### Statistical methods

In a stepped-wedge design clusters contribute different amounts of time to the intervention and control periods, making traditional measures of covariate balance between intervention arms difficult to use [44]. In this study, we will use a method to assess covariate balance by calculating a weighted average of each baseline characteristic for control and intervention periods; cluster [16] characteristics will be weighted by the amount of person-time they contributed to control and intervention periods (i.e., a cluster that crossed over in Step 2 contributed baseline covariates to two control periods (Steps 0–1) and three intervention periods (Steps 2–4)) [45].

Demographic and clinical characteristics of participants in the study will be described using means and standard deviations for continuous variables, and frequencies and percentages for categorical variables.

Primary and secondary analyses will be as randomised using a multi-level, mixed effects generalised linear model. The effect of the intervention and time period will be considered as fixed effects while the effect of treating general internal medicine unit and patient will be considered as random effects. This will include adjustment for block and time period of the design, seasonal variation in the outcomes (based on the previous 2 years of data), age and sex of the patient admissions [28, 30, 31].

We will make every effort to minimise missing outcome data at each wave. We will report the amounts of missing data according to published recommendations [46]. In addition, sensitivity analyses will be conducted to assess the robustness of the missing data assumption made in the primary analysis. A detailed analysis plan will be developed for secondary and sensitivity analyses. Statistical analyses will be carried out using Stata® (StataCorp. College Station, Texas USA).

### Economic analysis

Economic analysis and reporting will be consistent with published frameworks [47], including the Consolidated Health Economic Evaluation Reporting Standards (CHEERS) guidelines for reporting of health economic research [48]. The primary economic analysis within this study will analyse the difference in total direct costs in Australian dollars (at 2016 prices) from the health-service perspective. Total per-episode costings will be obtained, as well as individual item costs to identify the elements of patient care that underpin any significant cost differences between the two intervention models. Costings will include the 90 day post-discharge period to capture outpatient specialist, allied health, pathology and radiology services, as well as any readmission to hospital. Only costs incurred directly by Western Health will be included in this analysis.

A cost-effectiveness analysis from the health-service perspective will also be completed, with the incremental cost for each life year saved to be compared between the community-acquired pneumonia service best-practice intervention and usual care groups. This analysis will consider inpatient costs and mortality to 90 days following the original admission to hospital. Depending on the outcomes of preliminary analysis, appropriate analysis of uncertainty, such as use of non-parametric bootstrapping methods to calculate 95% confidence intervals with a corresponding cost-effectiveness plane and acceptability curves, will be explored [49].

### Data monitoring

Adverse events and adverse drug reactions that are considered to be associated with the delivery of the community-acquired pneumonia service protocolised interventions will be reported to the principal investigator within 72 hours. Patients receiving routine corticosteroids as a component of the community-acquired pneumonia service interventions will be reviewed daily and records will be kept of any hyperglycaemia requiring new insulin treatment. A log of adverse events and adverse drug reactions will be kept and reported to the project steering committee at quarterly meetings, and to the Melbourne Health Human Research Ethics Committee at 3 month intervals.

Serious adverse events and suspected unexpected serious adverse reactions will be reported to the Melbourne Health Human Research Ethics Committee within 72 hours. These may include (but are not limited to):

- Unexpected death
- Serious drug-related adverse event (directly linked to either antibiotic or corticosteroid dose) where it is required that the drug is stopped
- A fall with associated patient or clinician injury during physiotherapy
- Patient deteriorating clinically within 60 min of a physiotherapy session requiring a medical emergency team call or code blue (as defined by institutional procedures)

The project steering committee will meet quarterly to report enrolment numbers, protocol adherence or violations and adverse events and report these to the human research ethics committee. A data monitoring committee was not deemed necessary because outcome measures (e.g. length of stay) are (1) objective, (2) unambiguous and (3) collected through existing routine, validated measures (and therefore not prone to data integrity issues).

### Duration and timeline

It is anticipated that the study period will be completed in July 2017 and that data collection will be completed and analysed, and the manuscript prepared for submission, by December 2018. The final manuscript will be written in accordance with the relevant CONSORT statements and extensions [30].

Trial design and methods are summarised in Table 3.

**Table 3:** World Health Organization Trial Registration Data Set for IMPROVE-GAP trial

Data category	Information
Primary registry and trial identifying number	www.ClinicalTrials.gov, NCT02835040
Date of registration in primary registry	22 May 2016
Secondary identifying numbers	Not applicable
Trial protocol version	This is Version 3 of the protocol and was enacted on 24 August 2016
Source of monetary or material support	The HCF Research Foundation (AU $300,000)
Primary sponsor	The HCF Research Foundation
Secondary sponsor	Not applicable
Contact for public queries	HK, harin.karunajeewa@wh.org.au
Contact for scientific queries	HK, harin.karunajeewa@wh.org.au
Public title	IMPROVing Evidence-based treatment Gaps and outcomes in community-Acquired Pneumonia (IMPROVE-GAP).
Scientific title	IMPROVE-GAP: Evaluating the impact of a new model of care designed to improve evidence-based management of community-acquired pneumonia
Country of recruitment	Australia
Health condition or problem studied	Community-acquired pneumonia
Intervention	Active comparator: Evidence-based bundle of care (specifically: corticosteroids, early mobilisation, guideline-compliance antibiotic and early switch to oral antibiotic therapy, malnutrition risk screening and targeted nutritional therapy) Placebo comparator: Usual inpatient care
Key inclusion and exclusion criteria	Ages eligible for study: ≥ 18 yrs Sexes eligible for study: Both Accepts healthy volunteers: No Inclusion criteria: All adults admitted to the institution under a general internal medicine unit with community-acquired pneumonia (meeting a standardised community-acquired pneumonia definition) Exclusion criteria: (1) patients where a decision is made to implement palliative care on admission; (2) existing enrolment in a clinical trial.
Study type	Type: Investigator-initiated, interventional, pragmatic, study Allocation: Concealed randomisation Intervention model: Stepped-wedge rollout Masking: Patient and assessor blinded Primary purpose: Treatment Phase: Phase IV
Date of first enrolment	01/08/2016
Target sample size	Minimum 640 patients
Recruitment status	Recruiting
Primary outcome measure	Hospital length of stay
Key secondary outcome measures	Inpatient mortality, 30 and 90 day readmission rates and mortality, ICU admission and ventilation, total clinical costings, protocol adherence, serious adverse events

## Discussion

IMPROVE-GAP will be the first RCT powered and designed to investigate a service-delivery approach to improving the translation of evidence into clinical practice in community-acquired pneumonia, one of the highest-burden conditions affecting health systems worldwide. Its stepped-wedge design is better suited than other RCT approaches to evaluating a health-service intervention in a ‘real-world’ operational context. It also has pragmatic advantages in that once the study is completed, the intervention has effectively been implemented in the health system in which it has been tested and, should the implementation be found to be financially and clinically effective, can then be continued indefinitely. It may therefore demonstrate the power of stepped-wedge trial designs to accelerate the ‘time to translation’ when implementing clinical trial evidence in routine clinical practice [21]. This has value both to patients, who benefit from improved outcomes and, if the intervention also has an efficiency dividend, to the health system. We acknowledge the possibility that, despite considering patients masked to allocation owing to the waiver of consent, that it is possible that some patients were aware of study interventions. However, we feel that the risk of selection bias was minimised, as allocation to treating unit was determined by established practice according to day of admission or previous admitting unit. Our design also aimed to minimise attrition bias (by using routinely available outcome measures, such as length of stay, clinical cost and inpatient mortality) although readmission rate may be susceptible to attrition bias, as readmission to other health services was not routinely available nor collected.

An extremely important aspect of our study design was the decision by our supervising human research ethics committee to grant a waiver of consent for this study. This approach required very careful deliberation by both the investigating team and the human research ethics committee and represents an important precedent for conducting health-service research of this type, especially as it will allow enrolment of a study population that is truly representative of a ‘real-world’ scenario, generalisable to the population of patients with community-acquired pneumonia in developed countries and health care systems where most patients are now elderly, with high rates of multimorbidity and complex care needs. We believe this will be vital for high-quality health-service research in the future, by enabling a robust means of prospectively measuring the effectiveness of new health-service interventions in representative populations. We also strongly believe that this approach is ethically justifiable, based on its use of routinely available data, application of an intervention based on best practice (supported by a high level of existing evidence) and its concordance with current official ethical guidelines for use of a waiver of consent. Nonetheless, we acknowledge that the approach represents a departure from existing conventional clinical trial designs and could still be considered controversial. In particular, there can be subjectivity and disagreement within the clinical and scientific community as to the quality and applicability of evidence from previous clinical trials. This may undermine our assertion that our intervention represents ‘best practice’, which is fundamental to our argument of ethical defensibility. It is also notable that many aspects of our intervention that we deemed well-supported by evidence are yet to be incorporated into official consensus guidelines. In particular, the most widely cited community-acquired pneumonia management guidelines are those of the Infectious Diseases Society of America; these have not been updated since 2007. We decided, however, that these have been superseded by more recent studies and that the most ethically defensible approach therefore was to provide our patients with the benefit of knowledge accrued from large RCTs and meta-analyses performed more recently. However, we acknowledge that there was potential for subjective interpretation as to how we assessed the existing body of evidence in its entirety. It is also possible that the evidence on which we based our interventions will, in turn, be superseded by subsequent clinical trials. For these reasons, it is therefore possible that human research ethics committees in other settings and who operate under different guidelines might decide to rule against granting a waiver of consent for a study of this type. Regardless, IMPROVE-GAP represents an important precedent that should be a starting point for ongoing discussions regarding the best ways to prosecute future health-service research, particularly translation of best practice, in this ethically complex and challenging environment.

## Trial status

The trial is ongoing and is actively enrolling.

## Additional files

Additional file 1: SPIRIT 2013 Checklist. (DOC 123 kb)

Additional file 2: Detailed intervention description, [12, 14, 18, 39, 50, 51]. (DOCX 42 kb)

## Abbreviations

CHEERS: Consolidated Health Economic Evaluation Reporting Standards
CORB: Confusion (acute), oxygen saturation ≤ 90%, respiratory rate ≥ 30 breaths per minute, blood pressure < 90 mm Hg (systolic) or ≤ 60 mm Hg (diastolic)
PCR: polymerase chain reaction
RCT: Randomised controlled trial
REDCap: Research Electronic Data Capture
SpO_2_: pulse oximetry oxygen saturation
TIDieR: Template for Intervention Description and Replication

## References

[1] Global Burden of Disease Mortality Causes of Death Collaborators (2015). Global, regional, and national age-sex specific all-cause and cause-specific mortality for 240 causes of death, 1990–2013: a systematic analysis for the Global Burden of Disease Study 2013. Lancet.

[2] Arnold FW, Wiemken TL, Peyrani P, Ramirez JA, Brock GN, Authors C (2013). Mortality differences among hospitalized patients with community-acquired pneumonia in three world regions: results from the Community-Acquired Pneumonia Organization (CAPO) International Cohort Study. Respir Med.

[3] AIHW (2010). Asthma, chronic obstructive pulmonary disease and other respiratory diseases in Australia.

[4] The Australian Lung Foundation (2007). Respiratory infectious disease burden in Australia.

[5] Graf C (2006). Functional decline in hospitalized older adults: it’s often a consequence of hospitalization, but it doesn’t have to be. AJN Am J Nurs.

[6] Pulcini C, Couadau T, Bernard E, Lorthat-Jacob A, Bauer T, Cua E, Mondain V, Chichmanian RM, Dellamonica P, Roger PM (2008). Adverse effects of parenteral antimicrobial therapy for chronic bone infections. Eur J Clin Microbiol Infect Dis.

[7] Schindler M, Bernard L, Belaieff W, Gamulin A, Racloz G, Emonet S, Lew D, Hoffmeyer P, Uckay I (2013). Epidemiology of adverse events and *Clostridium difficile*-associated diarrhea during long-term antibiotic therapy for osteoarticular infections. J Infect.

[8] Valour F, Karsenty J, Bouaziz A, Ader F, Tod M, Lustig S, Laurent F, Ecochard R, Chidiac C, Ferry T, Lyon BJI Study Group (2014). Antimicrobial-related severe adverse events during treatment of bone and joint infection due to methicillin-susceptible *Staphylococcus aureus*. Antimicrob Agents Chemother.

[9] Weir DL, Majumdar SR, McAlister FA, Marrie TJ, Eurich DT (2015). The impact of multimorbidity on short-term events in patients with community-acquired pneumonia: prospective cohort study. Clin Microbiol Infect.

[10] Williams E, Girdwood J, Janus E, Karunajeewa H (2014). CORB is the best pneumonia severity score for elderly hospitalised patients with suspected pneumonia. Intern Med J.

[11] Torres A, Sibila O, Ferrer M (2015). Effect of corticosteroids on treatment failure among hospitalized patients with severe community-acquired pneumonia and high inflammatory response: A randomized clinical trial. JAMA.

[12] Blum CA, Nigro N, Briel M, Schuetz P, Ullmer E, Suter-Widmer I, Winzeler B, Bingisser R, Elsaesser H, Drozdov D (2015). Adjunct prednisone therapy for patients with community-acquired pneumonia: a multicentre, double-blind, randomised, placebo-controlled trial. Lancet.

[13] Popovic M, Blum CA, Nigro N, Mueller B, Schuetz P, Christ-Crain M (2016). Benefit of adjunct corticosteroids for community-acquired pneumonia in diabetic patients. Diabetologia.

[14] Siemieniuk RA, Meade MO, Alonso-Coello P, Briel M, Evaniew N, Prasad M, Alexander PE, Fei Y, Vandvik PO, Loeb M, Guyatt GH (2015). Corticosteroid therapy for patients hospitalized with community-acquired pneumonia: a systematic review and meta-analysis. Ann Intern Med.

[15] Marti C, Grosgurin O, Harbarth S, Combescure C, Abbas M, Rutschmann O, Perrier A, Garin N (2015). Adjunctive corticotherapy for community acquired pneumonia: a systematic review and meta-analysis. PLoS One.

[16] Mundy LM, Leet TL, Darst K, Schnitzler MA, Dunagan WC (2003). Early mobilization of patients hospitalized with community-acquired pneumonia. Chest.

[17] Ramirez JA, Vargas S, Ritter GW, Brier ME, Wright A, Smith S, Newman D, Burke J, Mushtaq M, Huang A (1999). Early switch from intravenous to oral antibiotics and early hospital discharge: a prospective observational study of 200 consecutive patients with community-acquired pneumonia. Arch Intern Med.

[18] Carratala J, Garcia-Vidal C, Ortega L, Fernandez-Sabe N, Clemente M, Albero G, Lopez M, Castellsague X, Dorca J, Verdaguer R (2012). Effect of a 3-step critical pathway to reduce duration of intravenous antibiotic therapy and length of stay in community-acquired pneumonia: a randomized controlled trial. Arch Intern Med.

[19] Bally MR, Yildirim PZB, Bounoure L, Gloy VL, Mueller B, Briel M, Schuetz P (2016). Nutritional support and outcomes in malnourished medical inpatients: a systematic review and meta-analysis. JAMA Intern Med.

[20] Adler N, Weber H, Gunadasa I, Hughes A, Friedman N (2014). Adherence to therapeutic guidelines for patients with community-acquired pneumonia in Australian hospitals. Clin Med Insights Circ Respir Pulm Med.

[21] Morris ZS, Wooding S, Grant J (2011). The answer is 17 years, what is the question: understanding time lags in translational research. J R Soc Med.

[22] Commonwealth of Australia. Medical Research Future Fund: Australian Medical Research and Innovation Priorities 2016–2018. http://health.gov.au/internet/main/publishing.nsf/Content/mrff. Accessed 13 Jan 2017.

[23] Hussain-Gambles M, Atkin K, Leese B (2004). Why ethnic minority groups are under-represented in clinical trials: a review of the literature. Health Soc Care Community.

[24] Mason S, Hussain-Gambles M, Leese B, Atkin K, Brown J (2003). Representation of South Asian people in randomised clinical trials: analysis of trials’ data. BMJ.

[25] Luce BR, Kramer JM, Goodman SN, Connor JT, Tunis S, Whicher D, Schwartz JS (2009). Rethinking randomized clinical trials for comparative effectiveness research: the need for transformational change. Ann Intern Med.

[26] Rothwell PM (2005). External validity of randomised controlled trials: “to whom do the results of this trial apply?”. Lancet.

[27] Lamb SE (2015). The case for stepped-wedge studies: a trial of falls prevention. Lancet.

[28] Haines TP, O’Brien L, Mitchell D, Bowles KA, Haas R, Markham D, Plumb S, Chiu T, May K, Philip K (2015). Study protocol for two randomized controlled trials examining the effectiveness and safety of current weekend allied health services and a new stakeholder-driven model for acute medical/surgical patients versus no weekend allied health services. Trials.

[29] Davey C, Hargreaves J, Thompson JA, Copas AJ, Beard E, Lewis JJ, Fielding KL (2015). Analysis and reporting of stepped wedge randomised controlled trials: synthesis and critical appraisal of published studies, 2010 to 2014. Trials.

[30] Hemming K, Haines TP, Chilton PJ, Girling AJ, Lilford RJ (2015). The stepped wedge cluster randomised trial: rationale, design, analysis, and reporting. BMJ.

[31] Hill AM, McPhail SM, Waldron N, Etherton-Beer C, Ingram K, Flicker L, Bulsara M, Haines TP (2015). Fall rates in hospital rehabilitation units after individualised patient and staff education programmes: a pragmatic, stepped-wedge, cluster-randomised controlled trial. Lancet.

[32] Foley N, Salter K, Teasell R (2007). Specialized stroke services: a meta-analysis comparing three models of care. Cerebrovasc Dis.

[33] Chan AW, Tetzlaff JM, Gotzsche PC, Altman DG, Mann H, Berlin JA, Dickersin K, Hrobjartsson A, Schulz KF, Parulekar WR (2013). SPIRIT 2013 explanation and elaboration: guidance for protocols of clinical trials. BMJ.

[34] Charles PG, Whitby M, Fuller AJ, Stirling R, Wright AA, Korman TM, Holmes PW, Christiansen KJ, Waterer GW, Pierce RJ (2008). The etiology of community-acquired pneumonia in Australia: why penicillin plus doxycycline or a macrolide is the most appropriate therapy. Clin Infect Dis.

[35] Charles PG, Wolfe R, Whitby M, Fine MJ, Fuller AJ, Stirling R, Wright AA, Ramirez JA, Christiansen KJ, Waterer GW (2008). SMART-COP: a tool for predicting the need for intensive respiratory or vasopressor support in community-acquired pneumonia. Clin Infect Dis.

[36] National Health and Medical Research Council. National statement on ethical conduct in human research. Canberra, Australia: NHMRC; 2007, updated 2015.

[37] Hoffmann TC, Glasziou PP, Boutron I, Milne R, Perera R, Moher D, Altman DG, Barbour V, Macdonald H, Johnston M (2014). Better reporting of interventions: template for intervention description and replication (TIDieR) checklist and guide. BMJ.

[38] Antibiotic Expert Groups (2014). Therapeutic guidelines: antibiotic. Version 15.

[39] Hodgson C, Needham D, Haines K, Bailey M, Ward A, Harrold M, Young P, Zanni J, Buhr H, Higgins A (2014). Feasibility and inter-rater reliability of the ICU mobility scale. Heart Lung.

[40] van Venrooij LM, de Vos R, Borgmeijer-Hoelen A, Kruizenga HM, Jonkers-Schuitema CF, de Mol B (2007). Quick-and-easy nutritional screening tools to detect disease-related undernutrition in hospital in-and outpatient settings: a systematic review of sensitivity and specificity. e-SPEN Eur E J Clin Nutr Metab.

[41] Watterson CFA, Banks M, Isenring E, Miller M, Silvester K, Hoevenaars R, Bauer J, Vivanti A, Ferguson M (2009). Evidence based practice guidelines for the nutritional management of malnutrition in adult patients across the continuum of care. Nutr Dietetics.

[42] Hemming K, Girling A (2014). A menu-driven facility for power and detectable-difference calculations in stepped-wedge cluster-randomized trials. Stata J.

[43] Harris PA, Taylor R, Thielke R, Payne J, Gonzalez N, Conde JG (2009). Research electronic data capture (REDCap) – a metadata-driven methodology and workflow process for providing translational research informatics support. J Biomed Inform.

[44] Brown CA, Lilford RJ (2006). The stepped wedge trial design: a systematic review. BMC Med Res Methodol.

[45] Gruber JS, Reygadas F, Arnold BF, Ray I, Nelson K, Colford JM (2013). A stepped wedge, cluster-randomized trial of a household UV-disinfection and safe storage drinking water intervention in rural Baja California Sur, Mexico. Am J Trop Med Hyg.

[46] Sterne JA, White IR, Carlin JB, Spratt M, Royston P, Kenward MG, Wood AM, Carpenter JR (2009). Multiple imputation for missing data in epidemiological and clinical research: potential and pitfalls. BMJ.

[47] Drummond M, Jefferson T (1996). Guidelines for authors and peer reviewers of economic submissions to the BMJ. BMJ.

[48] Husereau D, Drummond M, Petrou S, Carswell C, Moher D, Greenberg D, Augustovski F, Briggs AH, Mauskopf J, Loder E, ISPOR Health Economic Evaluation Publication Guidelines-CHEERS Good Reporting Practices Task Force (2013). Consolidated Health Economic Evaluation Reporting Standards (CHEERS) – explanation and elaboration: a report of the ISPOR Health Economic Evaluation Publication Guidelines Good Reporting Practices Task Force. Value Health.

[49] Briggs AH, O’Brien BJ, Blackhouse G (2002). Thinking outside the box: recent advances in the analysis and presentation of uncertainty in cost-effectiveness studies. Annu Rev Public Health.

[50] Berney S, Haines K, Skinner EH, Denehy L (2012). Safety and feasibility of an exercise prescription approach to rehabilitation across the continuum of care for survivors of critical illness. Phys Ther.

[51] Lizarondo L, Kumar S, Hyde L, Skidmore D (2010). Allied health assistants and what they do: a systematic review of the literature. J Multidiscip Healthc.

