# Supplementary material for: The IMPROVE-GAP Trial aiming to improve evidence-based management of community-acquired pneumonia: study protocol for a stepped-wedge randomised controlled trial
Source: Trials. 2018 Feb 5;19:88. doi: 10.1186/s13063-017-2407-4 (PMC5800278; doi:10.1186/s13063-017-2407-4)
Supplement: Supplementary file 2 — Detailed intervention description, [12, 14, 18, 39, 50, 51]. (DOCX 42 kb) [file 13063_2017_2407_MOESM2_ESM.docx]

**Additional File 2 – Detailed intervention description**

**Intervention 1 – Corticosteroids**

Participants with specific contraindications will not receive this intervention [[12](#_ENREF_12), [14](#_ENREF_14)]:

- active intravenous drug use;
- acute burn injury or gastrointestinal bleeding in the past 3 months;
- known adrenal insufficiency;
- pregnancy or breastfeeding;
- a concurrent condition requiring >0.5mg/kg prednisolone per day; and
- severe immunosuppression defined as one of the following: infection with human immunodeficiency virus and a CD4 cell count below 350 cells per μL, immunosuppressive therapy after solid organ transplantation, neutropenia below 500 cells per μL or neutrophils of 500–1000 cells per μL during ongoing chemotherapy with an expected decrease to values below 500 cells per μL, cystic fibrosis, or active tuberculosis [[12](#_ENREF_12)].

**Intervention 2 – Early switch to oral antibiotics**

Patients will be switched from intravenous to oral therapy according to the following criteria for clinical improvement assessed on a daily basis by the CAP Service using a standardised check-list [[18](#_ENREF_18)] as follows:

- ability to maintain oral intake;
- stable vital signs (temperature ≤37.8 degrees Celsius; respiratory rate ≤24 breaths/min; systolic blood pressure ≥90mm Hg without vasopressor support for at least 8 hours);
- stable SpO_2_ > 92% without supplementary oxygen (> 88% for patients with known respiratory disease where a modification is documented on the observation chart), unless the patient was previously receiving home oxygen; and
- absence of septic metastases/major exacerbated co-morbidities

**Intervention 3 – Early mobilisation**

Progressive movement will be defined as achieving a higher score on the ICU Mobility Scale when compared to the preceding day, or an increase in the total distance mobilised during the physiotherapy session. The patient mobilisation intervention will be progressed daily by a physiotherapist, or allied health assistant under physiotherapist direction, with the aim of achieving the patient’s pre-morbid mobility level and exercise tolerance as soon as possible. Premorbid mobility will be categorised according to the ICU Mobility Scale which has previously been used in hospital inpatients [[39](#_ENREF_39)].

Participants with the following specific contraindications (assessed daily) will not receive this intervention [[50](#_ENREF_50)]:

- Systolic blood pressure >180 or <90, OR ≤10mm Hg than physiological baseline systolic or diastolic pressure in patients with pre-existing renal disease;
- Heart rate <50 or >110 bpm (if abnormal for patient) +/- new arrhythmia;
- Vasopressor support;
- Respiratory rate >25 breaths per minute;
- Fraction of inspired oxygen (FiO_2_) >0.6 with arterial partial pressure of oxygen (PaO_2_) <70 mm Hg OR oxygen saturation (SpO_2_) <90% (<88% for patients with respiratory disease where a modification is documented on the observation chart);
- Acute clinical deterioration since last mobilisation session;
- Participant drowsy and unable to follow commands;
- Recent fall not yet assessed by medical staff; or
- New onset chest pain.

During daily mobilisation, the listed observations will be measured if clinically indicated, and the patient will be directed to take a seated rest should any of the observations deviate as follows:

- Systolic blood pressure >180 or <90, OR ≤10mm Hg than physiological baseline systolic or diastolic pressure in patients with pre-existing renal disease;
- Decrease in SpO_2_ >10% or SpO_2_ <85% during mobilisation;
- Respiratory rate > 25;
- Heart rate <50 or >110 bpm (if abnormal for patient); and/or
- Patient becomes breathless and unable to speak in sentences.

If any of the above occur but recover to within normal limits within two minutes, the patient will continue with prescribed mobilisation. If the patient does not recover within two minutes, early mobilisation will cease, the patient’s nurse will be notified and asked to monitor the patient, and relevant observations will be documented in the patient’s medical record.

In the event any of the following adverse events occur during mobilisation an urgent medical review, Medical Emergency Team call or Code Blue will be activated in accordance with institutional procedures:

- New onset chest pain or patient becomes pale and sweaty, drowsy or has a change in conscious state,
- Sustained (after >5 minutes rest):
  - Systolic blood pressure >180 or <90 OR ≤10mm Hg than normal systolic or diastolic pressure in patients with renal disease, or
  - Heart rate <50 or >140 (if abnormal for patient), or
  - SpO_2_ <90% (<88% for patients with respiratory disease where a modification is documented on the observation chart) despite appropriately titrated oxygen therapy, or
  - Respiratory rate >25.
- Patient has a fall with associated injury.

Early mobilisation will continue daily until the patient meets the CAP intravenous antibiotic stopping rules (listed in Intervention 2), and one of:

1. Patient is discharged home; or
2. Patient achieves self-reported pre-morbid functional status and exercise tolerance. In this case the patient will be referred to the general functional maintenance program delivered by an Allied Health Assistant [[51](#_ENREF_51)] daily whilst an acute inpatient; or
3. Patient is medically stable for transfer to inpatient rehabilitation or a sub-acute aged care setting; or
4. Patient refuses to participate in early mobilisation for three consecutive days. In these cases, the patient will be referred back to the physiotherapist assigned to the relevant GIM unit for any further management.
